# Vitamin D supplementation for depression in older adults: a meta-analysis of randomized controlled trials

**DOI:** 10.3389/fnut.2023.1169436

**Published:** 2023-06-21

**Authors:** Yoonjung Park, Young-Mi Ah, Yun Mi Yu

**Affiliations:** ^1^Department of Industrial Pharmaceutical Science, Yonsei University, Incheon, Republic of Korea; ^2^Department of Pharmacy and Yonsei Institute of Pharmaceutical Sciences, College of Pharmacy, Yonsei University, Incheon, Republic of Korea; ^3^College of Pharmacy, Yeungnam University, Gyeongsan, Republic of Korea

**Keywords:** geriatrics, older adults, vitamin D, vitamin D supplementation, depression, depressive symptoms

## Abstract

**Background:**

In older adults, depression is associated with several other clinical problems such as cognitive impairment and low quality of life. Several studies have evaluated the relationship between vitamin D and depression in older adults; however, the results have been controversial thus far.

**Objective:**

This study aimed to investigate the effects of vitamin D supplementation on depressive symptom improvement among individuals aged ≥60 years with or without a diagnosis of depression or depressive symptoms based on a meta-analysis of randomized controlled trials (RCTs).

**Methods:**

RCTs were identified to analyze the relationship between vitamin D supplementation and depressive symptoms. MEDLINE, CENTRAL, Embase, and PsycINFO were systematically searched for relevant articles published from inception to November 2022. RCTs that evaluated the effect of vitamin D supplementation in participants aged ≥60 years compared to placebo were included. A random effects model was used in this meta-analysis because of the differences between the included RCTs. The quality of the RCTs was assessed using Risk of Bias 2.

**Results:**

Seven trials were included in the analyses. The primary outcome of pre-post score changes included five trials with a total of 752 participants. The secondary outcome of post-intervention score included all seven trials with a total of 4,385 participants. No significant improvement in depressive symptoms in either pre-post score changes [standardized mean difference (SMD) = −0.49; 95% confidence interval (CI) −1.07–0.09; *p* = 0.10] or post-intervention score (SMD = −0.10; 95% CI −0.28–0.07; *p* = 0.25) was found.

**Conclusion:**

Vitamin D supplementation in older adults was not associated with an improvement in depressive symptoms. More studies in older adults are needed to evaluate the association between vitamin D supplementation and depression.

## 1. Introduction

The increasing number of older adults worldwide presents a challenge for the health and social systems. The pace of the demographic shift is accelerating, with an estimated population of older adults of 2.1 billion by 2050 (1). The gradual decline in mental and physical capacity increases the occurrence of chronic diseases, forming complex health conditions called geriatric syndromes (1). The estimated prevalence of depression differs by age; however, WHO estimated the depression in older adults to range between 10 and 20% (2). Depression is often underdiagnosed and undertreated owing to the co-existence of other health problems that may increase the risk of cognitive impairment, suicidal thoughts, and low quality of life (3, 4). Multiple risk factors other than co-existing health problems may exacerbate depression in older adults, such as a decline in daily activities, changes in socioeconomic status, chronic physical pain, and loss of a loved one (3, 5). Moreover, struggles with many life stressors may induce isolation and loneliness in older adults (3).

Various safe and effective pharmacotherapies are available for treating depression. Medications such as selective serotonin reuptake inhibitors, tricyclic antidepressants, and antipsychotics are widely administered to control depressive symptoms (6). For optimal treatment, many factors must be considered, such as drug metabolism, pharmacokinetic and pharmacodynamic changes in older adults, drug–drug interactions with concurrent medications, and side effect profiles (5–7). Although monotherapy is preferred to minimize adverse effects and increase compliance, only 40 percent of the patients respond fully to the first agent (6). Non-responders need to change to a different class or add a second agent to achieve benefits (6). However, susceptibility to side effects is a concern for older adults owing to concurrent medications and diseases (7). Therefore, an adjuvant therapy that is safer and helps control depressive symptoms could be a good option for older adult patients.

The role of vitamin D has expanded beyond calcium and bone health, as a result of the discovery of vitamin D receptors (VDRs) in the brain, particularly in the hypothalamus (8, 9), which is involved in the pathophysiology of depression. In the brain, vitamin D_3_ is converted into its active form (1,25(OH)D) by 1-alpha-hydroxylase (8), which may be associated with neuroprotective properties (10). The imbalance of monoamine neurotransmitters such as serotonin, dopamine, and norepinephrine in the brain is closely related to depression (11). Vitamin D deficiency can decrease the synthesis of these neurotransmitters and downregulate the gene expression of tyrosine hydroxylase, an enzyme involved in the synthesis of dopamine and norepinephrine, leading to the development of depression (11–13). A recent *in vivo* study suggested that 1,25(OH)D mimics antidepressants by inhibiting serotonin reuptake and the gene expression of monoamine oxidase-A in cultured rat serotonergic neuronal cell lines (14). Despite supporting evidence from preclinical studies demonstrating the role of vitamin D in depression, the efficacy of vitamin D supplementation in the older population remains unclear.

Although vitamin D can be endogenously produced through the skin by sun exposure and obtained from dietary sources, vitamin D deficiency is common in older adults mainly due to reduced nutritional intake, limited outdoor activities, and poor skin integrity (15). Vitamin D intake from food is limited owing to few naturally-occurring vitamin D-rich food sources (16). In addition, Irandoust et al. (12) reported that incorporating vitamin D supplementation with physical activity could lead to greater improvements in vitamin D levels and depressive symptoms among women with obesity and depression compared to physical activity alone. Considering these aspects, vitamin D supplementation in older adult patients may be a safe and cost-effective adjuvant therapy for depressive symptom control. Vitamin D supplementation for older adults is recommended because vitamin D deficiency is closely related to depression. Recent evidence suggests that vitamin D deficiency causes a higher burden of depressive symptoms (17), and older patients with depression have low levels of vitamin D (18). In recent decades, many studies have evaluated the relationship between vitamin D and depression, especially in adults; however, the results are controversial. Previous systematic reviews and meta-analyses in adults showed no effects in alleviating depressive symptoms (19, 20). Recently, Albuloshi et al. (13) found that high-dose vitamin D supplementation reduced depressive symptoms in comparison to low vitamin D doses in adults aged ≥18 years. Some randomized controlled trials (RCTs) have demonstrated the positive effects of vitamin D supplements on depression (21, 22), whereas others showed no effects (23, 24). In their recent meta-analysis, Li et al. reported an association between depression and serum vitamin D levels in older adults; however, they did not evaluate the effectiveness of vitamin D supplementation (25). Although there are many studies published on the current topic, none of the systematic reviews and meta-analyses of RCTs addressed the efficacy of vitamin D supplementation on depression in older adults. Therefore, this study aimed to perform a meta-analysis to evaluate the efficacy of vitamin D in improving depressive symptoms in older adult patients.

## 2. Methods

This study followed the guidelines recommended by the Preferred Reporting Items for Systematic Reviews and Meta-Analyses (PRISMA 2020) (Supplementary Table S1) (26). The study protocol is available from the PROSPERO database (CRD42022311841). Two authors (YP and YMY) independently performed the literature search, study selection, data extraction, and bias assessment. A third investigator (Y-MA) resolved any discrepancies between authors.

### 2.1. Search strategy

The MEDLINE, Embase, CENTRAL, and PsycINFO databases were searched for RCTs that evaluated the association between vitamin D supplementation and depressive symptoms. Relevant studies and search terms were explored from inception to November 30, 2022, using medical subject heading on each database as follows: “aged,” “vitamin D,” “depression,” and “randomized controlled trials.” The following keywords were searched in the databases to identify eligible studies: “aged,” “elder*,” “senior,” “older people,” “later life,” “vitamin D,” “vitamin D*,” “cholecalciferol*,” “calciferol*,” “depression,” “depressive disorder,” “involutional psychoses,” “involutional melancholia,” “randomized controlled trial,” “controlled clinical trial,” and “trial.” The complete search strategy is presented in Supplementary Table S2.

### 2.2. Study selection

Studies were considered eligible if they met the following criteria: (1) population: participants aged ≥60 years with or without depression; (2) intervention: administration of vitamin D supplements; (3) comparison: placebo therapy; (4) primary and secondary outcomes: pre-post score changes and post-intervention score; and (5) study design: RCTs. The following studies were excluded: (1) non-human studies, including animal and *in vitro* studies; (2) non-randomized studies or case reports; (3) reviews, meta-analyses, or ongoing studies; (4) studies available only in the form of abstracts or posters; and (5) studies published in languages other than English.

### 2.3. Data extraction and quality assessment

Eligible studies were reviewed, and the following data were extracted using a standardized extraction form: first author, publication year, country, inclusion criteria, vitamin D dosing regimen, depression assessment scales, follow-up duration, number of participants, age, sex, baseline level of vitamin D, number of patients with current depression, and depression scores. Serum concentrations of vitamin D in nanomoles per liter (nmol/L) were converted into nanograms per milliliter (ng/mL) (27). Additionally, vitamin D dosage units were converted into international units per day (IU/day) to ensure data consistency.

The Risk of Bias 2 (ROB 2) tool (28) was used to conduct a quality assessment of each included RCT. The tool is structured into five domains of bias as follows: (1) bias arising from the randomization process; (2) bias due to deviations from the intended interventions; (3) bias due to missing outcome data; (4) bias in the measurement of the outcome; and (5) bias in the selection of the reported results. The judgement of risk of bias was based on responses to all questions in each domain (28). The overall risk of bias was evaluated as low, high, or some concerns. Any disagreements were resolved through discussion.

### 2.4. Study outcomes

The primary study outcome was to evaluate the pre-post score changes (change of scores from the baseline) by pooling standardized mean difference (SMD). The post-intervention SMD was evaluated as a secondary outcome to find the association between vitamin D supplementation and depressive symptom improvement. The following depression assessment scales were used to measure depressive symptoms: Center of Epidemiological Studies Depression Scale (CES-D), a score of 16 or greater was an indication of depressive symptoms; Geriatric Depression Scale – 15 (GDS-15), a score of 5 or greater indicated mild to moderate depression, and a score of 10 or greater indicated severe depression; 9-Item Patient Health Questionnaire Depression Scale (PHQ-9), more depressive symptoms were presented with a higher score ranging between 0 (no significant depressive symptoms) and 24 points (severe depressive symptoms); 12-Items Short Form Health Survey (SF-12) mental component score (MCS), which indicated depression if the score was ≤42 or less; and 21-Item Depression Anxiety and Stress Scale (DASS-21), a score between 5 and 6 indicated mild depression, a score between 7 and 10 indicated moderate depression, and a score between 11 and 13 indicated severe depression.

### 2.5. Statistical analysis

Statistical analysis was performed using the Cochrane systematic review software Review Manager (RevMan) [Computer program]. Version 5.4, The Cochrane Collaboration, 2020. The pooled SMD with 95% confidence intervals (CIs) of the pre-post changes and post-intervention were computed using the generic inverse variance method (29). The pre-post changes of mean difference were calculated from the difference between the placebo and intervention groups. The standard deviations (SDs) of the mean changes from baseline were calculated imputing a correlation coefficient (*Corr*) of 0.7 based on previous literature (30, 31) because SDs were not reported in most studies. The formula for calculating the SDs is as follows (32):


$$SD_{\mathrm{change}}=\sqrt{SD^{2}{}_{\mathrm{baseline}}+SD^{2}{}_{\mathrm{final}}-\left(2\times Corr\times SD_{\mathrm{baseline}}\times SD_{\mathrm{final}}\right)}$$

*SD*_change_ represents the SD of the mean changes from baseline, *SD*_baseline_ is the SD of before treatment, *SD*_final_ corresponds to the SD of post-intervention, and *Corr* represents the correlation between the baseline and final measurements. Baseline and final SDs were provided in each included study. Therefore, the *SD*_change_ values were calculated by assigning a *Corr* value of 0.7 in the equation. The post-intervention SMD was calculated from the depression score after vitamin D supplementation between the intervention and placebo groups. Heterogeneity was assessed using Cochrane’s Q and *I^2^* statistics, with significance set at *p* > 0.1 and *I^2^* > 50% (33). A fixed-effects model was used in the absence of significant heterogeneity, and a random-effects model was used in the presence of significant heterogeneity (34).

The subgroup analysis was conducted to evaluate the difference between the treatment and placebo groups by potential factors. The subgroup analyses were stratified by baseline vitamin D level, daily dose of vitamin D, and follow-up duration using pre-post score changes and post-intervention score. Female-only subgroup analysis was only conducted in post-intervention score due to the limited number of eligible studies. Publication bias was not examined because the number of included studies was <10.

## 3. Results

### 3.1. Study selection

**Figure 1:**
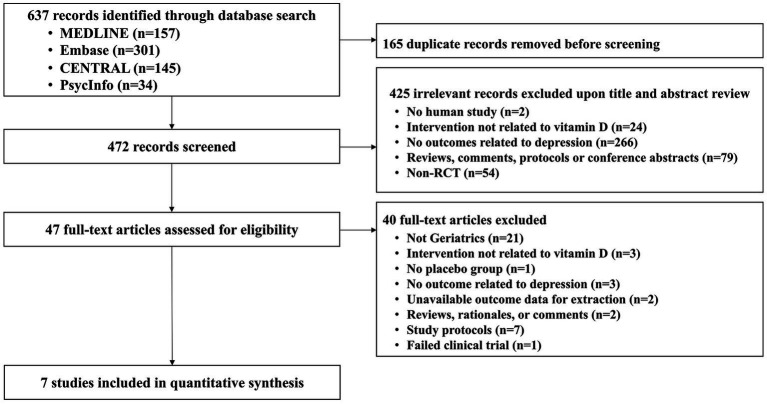
Flow chart of the study selection process.

A total of 637 articles were identified through an electronic database search and 165 duplicated articles were removed. Next, 472 articles were screened for relevance based on their titles and abstracts, resulting in the exclusion of 425 articles. The remaining 47 articles were assessed for eligibility through full-text evaluation, and 40 were excluded. Finally, seven RCTs (35–41) with 4,385 participants were selected for quantitative synthesis (Figure 1).

### 3.2. Study characteristics

Table 1 summarizes the characteristics of the seven included RCTs (35–41). Two studies were conducted in Australia (38, 40), while the others were conducted in the Netherlands (36), the United States of America (39), Iran (35), Greece (41), and the United Kingdom (37). The number of participants ranged from 78 to 2,012. Oral vitamin D_3_ (cholecalciferol) was administered in all studies, except for that by Yalamanchili et al. (39) who administered oral calcitriol, the most active form of vitamin D. The dose of vitamin D_3_ varied from 600 to 7,143 IU/day. The follow-up duration ranged from 8 weeks to 5 years.

**Table 1:** Characteristics of included studies.

First author, publication year	Country	Study population	Sample size^a^		Vit. D regimen (daily dose IU/day)	Depression assessment scale	Follow-up duration
			Vit. D	Placebo			
Alavi, 2019	Iran	Adults ≥60 y under treatment for depression and no hx of other mental illness	39	39	Vit D3, 50,000 IU/wk. (7,143)	GDS-15	8 wk
De Koning, 2019	Netherlands	Community-dwelling adults with depressive symptoms	75	76	Vit D3, 1,200 IU/day	CES-D	12 mo
Dumville, 2006	United Kingdom	Women ≥70 y with one or more risk factors for hip fracture	680	941	Vit D3, 800 IU/day	SF-12	6 mo
Sanders, 2011	Australia	Adults ≥70 y with an identified risk factor for hip fracture	1,001	1,011	Vit D3, 500,000 IU/yr. (1,370)	SF-12	3–5 y
Yalamanchili, 2012	USA	Community-dwelling postmenopausal women	123	123	Calcitriol, 0.5 g/day (20 million IU)	GDS-LF30	36 mo
Zajac, 2020	Australia	Community-dwelling healthy older adults	89	92	Vit D3, 600 IU/day	DASS-21	6 mo
Zaromytidou, 2022	Greece	Adults >60 y diagnosed with prediabetes according to ADA	42	35	Vit D3, 25,000 IU/wk. (3,571)	PHQ-9	12 mo

a Number of patients who completed the study.

ADA, American Diabetes Association; CES-D, Center for Epidemiologic Studies Depression Scale; DASS-21, Depression Anxiety Stress Scales-21; GDS-15, Geriatric Depression Scale-15; GDS-LF30, Geriatric Depression Scale-Long Form 30; hx, history; mo, months; PHQ-9, Patient Health Questionnaire; SF-12, 12-Item Short Form Survey; Vit., vitamin; wk, weeks; y, years.

The baseline characteristics of the study population are presented in Table 2. The mean age of participants in the studies varied from 67.9 to 76.8 years. Women accounted for more than half of the participants in all studies, of which three studies (37–39) recruited only women. The mean vitamin D levels ranged from 17.5 to 31.2 ng/mL. Regarding the current depression status, only Zajac et al. (40) excluded participants with depression, three studies (35, 36, 39) included participants with depression, and the other three studies (37, 38, 41) did not specify.

**Table 2:** Baseline characteristics of the study population.

First author, year	Mean age, mean ± SD, years		Women, %		Vit. D level, mean ± SD, ng/mL		Current depression, %	
	Vit. D	Placebo	Vit. D	Placebo	Vit. D	Placebo	Vit. D	Placebo
Alavi, 2019	68.7 ± 7.0	67.0 ± 6.3	48.7	51.3	22.6 ± 6.2	21.2 ± 5.8	100.0	100.0
De Koning, 2019	67.8 (65.4–71.7)^a^	67.3 (63.4–72.0)^a^	58.4	56.4	18.4 (13–22.8)^a^	17.6 (14.4–22.1)^a^	100.0^b^	100.0^b^
Dumville, 2006	77.0 ± 5.1	76.7 ± 4.9	100.0	100.0	NA	NA	NA	NA
Sanders, 2011	75.8 (72.9–79.9)^a^	75.9 (72.9–79.2)^a^	100.0	100.0	NA	NA	NA	NA
Yalamanchili, 2012	71.8 ± 3.4	71.1 ± 3.7	100.0	100.0	30.6 ± 9.4	31.7 ± 11.0	9.8^c^	13.8^c^
Zajac, 2020	70.8 ± 6.4	70.1 ± 5.7	53.8	51.1	31.1 ± 0.8	29.7 ± 0.8	0^d^	0^d^
Zaromytidou, 2022	73.1 ± 7.2	74.0 ± 7.6	80.0	77.8	19.98 ± 6.73	19.85 ± 5.72	NA	NA

a Median (interquartile range).

b Includes participants with depressive symptoms based on a CES-D scale score ≥ 16.

c Found women had depression based on baseline GDS score.

d Excludes participants with depression based on the CES-D score of ≥ 16.

### 3.3. Improvement of depressive symptoms

**Figure 2:**
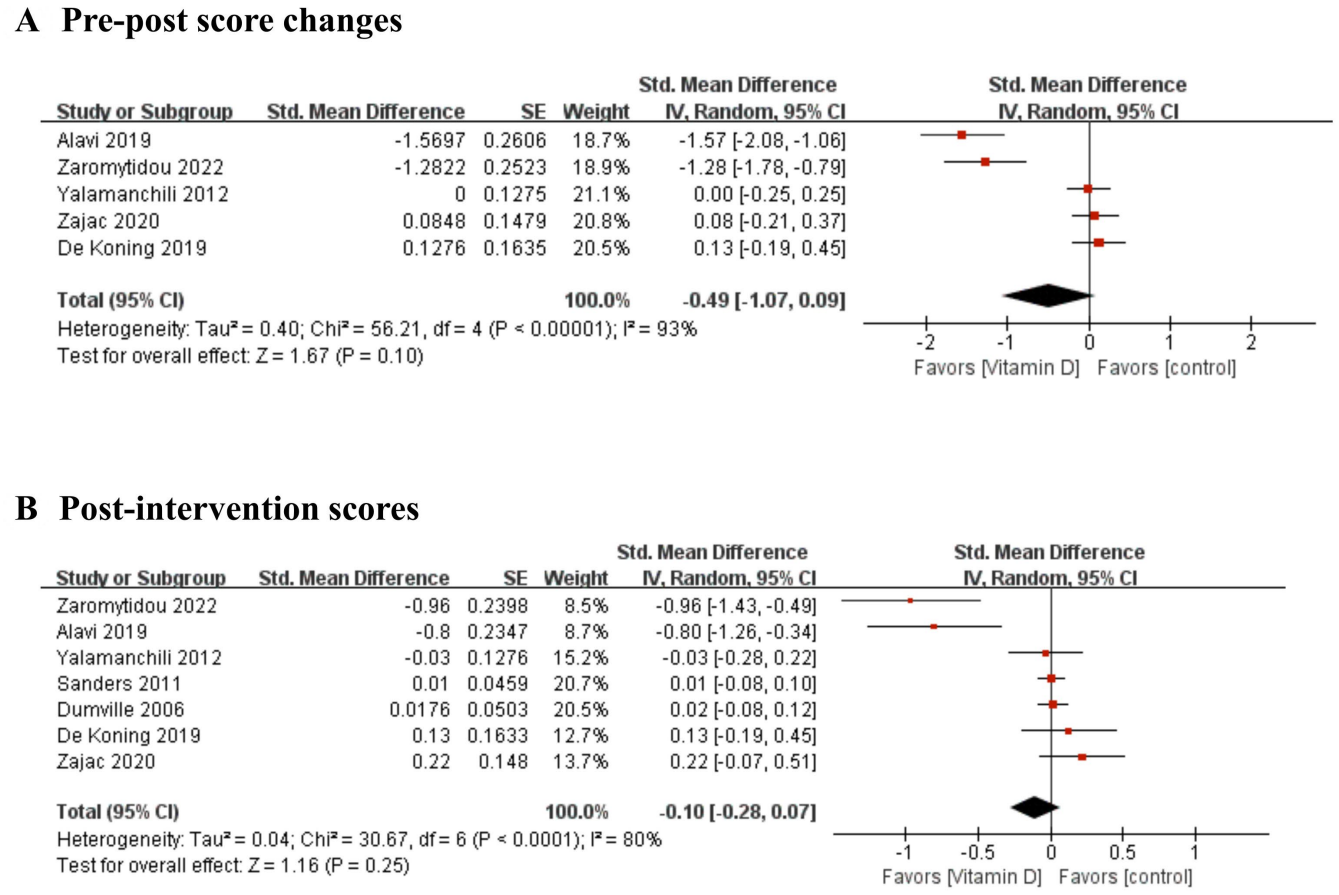
Forest plot of the improvement of depressive symptoms. **(A)** pre-post score changes and **(B)** post-intervention scores.

The primary outcome of pre-post score changes included five studies (35, 36, 39–41) involving 752 participants. Two studies (37, 38) were excluded from pre-post score changes because one study (38) did not report baseline depression score and the other study (37) only reported baseline and the SMD of post-intervention score. The secondary outcome of post-intervention score included all seven studies (35–41) involving 4,385 participants. No significant difference was found in either pre-post score changes (SMD = −0.49; 95% CI -1.07–0.09; *p* = 0.10; *I^2^* = 93%) or post-intervention score (SMD = −0.10; 95% CI -0.28–0.07; *p* = 0.25; *I^2^* = 80%), although the pooled analysis showed a trend of favoring the vitamin D group (Figure 2).

No statistically significant differences were observed in subgroup analyses of either pre-post score changes or post-intervention score. The subgroup analysis was stratified according to baseline vitamin D level < 30 ng/mL, vitamin D administration dose ≤1,500 and > 1,500 IU/day, and follow-up years ≤1 and > 1 year. Female-only subgroup analysis was only conducted in post-intervention score because of the limited number of eligible studies analysis. The results of the subgroup analysis are shown in Table 3.

**Table 3:** Subgroup analysis of pre-post score changes and post-intervention scores.

Variables	Number of studies (sample size)	Pooled SMD (95% CI)	*I^2^* (%)	*p*-value^a^
*Pre-post intervention*				
Baseline Vit D level < 30 ng/mL	3 (323)	−0.89 (−2.03–0.25)	95	0.12
Vit D daily dose				
≤ 1,500 IU/day	2 (338)	0.10 (−0.11–0.32)	0	0.07
> 1,500 IU/day	3 (414)	−0.93 (−2.02–0.15)	95	
Follow-up duration				
≤ 1 year	4 (506)	−0.64 (−1.44–0.17)	94	0.14
> 1 year	1 (246)	0.00 (−0.25–0.25)	93	
*Post-intervention*				
Baseline Vit D level < 30 ng/mL	3 (323)	−0.52 (−1.24–0.20)	90	0.16
Vit D daily dose				
≤ 1,500 IU/day	4 (3971)	0.03 (−0.04–0.09)	0	0.07
> 1,500 IU/day	3 (414)	−0.57 (−1.20–0.07)	88	
Follow-up duration				
≤ 1 year	5 (2127)	−0.23 (−0.59–0.13)	88	0.21
> 1 year	2 (2258)	0.01 (−0.08–0.09)	0	
Female-only	3 (3879)	0.01 (−0.05–0.07)	0	0.75

a *p*-value for subgroup differences.

Significance level < 0.05. CI, confidence interval; SMD, Standardized mean difference; Vit, vitamin.

### 3.4. Quality assessment

The risk of bias assessment revealed that among the seven included studies, two (37, 41) had a high-risk of bias owing to missing data and open-label design (Supplementary Figure S1; Supplementary Table S3).

## 4. Discussion

This meta-analysis evaluated the efficacy of vitamin D supplementation on depressive symptoms compared to placebo in older adults. To the best of our knowledge, this is the first meta-analysis to evaluate the efficacy of vitamin D supplementation on depressive symptoms in older adults. No significant differences were detected between the vitamin D supplementation and placebo groups in either pre-post score changes or post-intervention score. In addition, clinical significance could be limited due to minimal differences. Although previous meta-analyses included adult participants of all ages, our findings were congruent with those of Li et al. (19) and Gowda et al. (20) who reported that vitamin D supplementation had no significant effect on depressive symptoms. Although the pooled analysis showed no significant effects in improving depressive symptoms, two individual studies (35, 41) showed distinct effects favoring the vitamin D group (Figure 2). The vitamin D dosage in these two studies was higher than that in other studies, and they included the participants with mean baseline vitamin D levels <30 ng/mL. Considering these aspects of studies, future trials should consider administering high doses of vitamin D and including only participants with vitamin D deficiency.

No notable outcomes were found in all subgroup analyses. Insufficient RCTs were available to dichotomize baseline 25(OH)D into sufficient and deficient levels; however, a potential of positive effects of vitamin D supplementation was shown in vitamin D-deficient patients without reaching statistical significance (SMD = −0.89; 95% CI -2.03–0.25; *p* = 0.12; *I^2^* = 95%). Considering that vitamin D deficiency is a known risk factor for depression (15), additional studies are needed to evaluate the preventive effects of vitamin D supplementation in older adults. Although no differences were found in the subgroups stratified by the dose of ≤ or > 1,500 IU/day, the participants who received a dose >1,500 IU/day showed a tendency for depressive symptom improvement more than those who received ≤1,500 IU/day. This finding is congruent with the American Endocrine Society recommendation of vitamin D doses between 1,500 and 2000 IU/day to maintain vitamin D adequacy (> 30 ng/mL) (42). The participants in two (35, 41) of the three studies that administered high-dose vitamin D were classified as having a baseline vitamin D level of <30 ng/mL. This agrees with the results of a recent meta-analysis that demonstrated that high-dose vitamin D administration (≥ 4,000 IU) significantly improved depressive symptoms in adults (13). The methodological heterogeneity of the included studies, such as variations in vitamin D dose (daily vs. monthly), route of administration (tablets vs. bolus), duration of the vitamin D supplementation period, depression status of participants, depression scales used, and baseline vitamin D levels, may have contributed to the lack of differences between the intervention and placebo groups. We could not perform an analysis by the aim of vitamin D supplementation, such as the treatment or prevention of depression, because the number of studies was limited. Follow-up years of both pre-post changes and post-intervention did not show a significant difference. Moreover, no significant gender difference was found in subgroup analysis using post-intervention scores. Considering the results of individual studies, vitamin D in healthy older adults had no effect (40). In patients with depression, the results were inconclusive because one study reported a positive outcome (35), whereas another reported no effect (36). The subgroup findings with small marginal differences may cause uncertainty and should be interpreted with caution.

Growing evidence suggests possible mechanisms of vitamin D in mood regulation. The active form of vitamin D is produced in the brain by 1-alpha-hydroxylase in the vicinity of the VDRs (8). The binding of active vitamin D to VDRs in the hippocampus is involved in antioxidant, and anti-inflammatory activities, production of neurotrophic factors, and biosynthesis of monoamines, which are responsible for the regulation of mood changes (10). Another proposed mechanism relates to the homeostasis of calcium, which regulates the equilibrium between glutamate, an excitatory neurotransmitter, and GABA, an inhibitory neurotransmitter (43). The imbalance of these neurotransmitters can impact the onset of depression. In addition, vitamin D intake in older patients is often insufficient. The average intake of vitamin D from beverages and food is only 347.05 ± 307.8 IU, which is well below the recommend daily intake for adults (44). Cutaneous production of vitamin D_3_ is affected by time of day, latitude, aging, sunscreen use, and degree of skin pigmentation (45). Limited outdoor physical activities and exercise due to restricted mobility in older adults reduces sun exposure and decreases cutaneous synthesis of vitamin D (15). Given the role of vitamin D in depression and the likelihood of vitamin D deficiency among older patients, it is important to determine the optimal dosage for treating depressive symptoms in this population.

Inconclusive outcomes may be due to methodological issues and the characteristics of vitamin D that differentiate it from conventional drugs (46, 47). Vitamin D level is associated with the seasons, physical activity, eating habits, and cutaneous synthesis of vitamin D; however, we could not evaluate the effects of these factors (15). In addition, some studies reported that the post-vitamin D level in the control group was higher than that at baseline, which might be associated with the aforementioned factor (35, 41). Therefore, the effects of vitamin D supplementation could be underestimated owing to the lack of control over the confounding factors. Additional well-designed studies are required to evaluate the effects of vitamin D on depression in older adults.

This study has several limitations. First, the number of studies included in the meta-analysis was small and heterogeneous with respect to several factors, such as the vitamin D supplementation regimen, baseline vitamin D level, and depression status. Thus, observing a meaningful change in depressive symptom improvement with vitamin D supplementation can be difficult. We performed subgroup analyses to identify more meaningful changes to minimize our limitations. Not all studies were included in the subgroup analysis due to missing information for further investigation. Physical status, including comorbidities and disabilities, as well as psychosocial factors and lifestyle, are potentially significant factors contributing to depression among older patients and should be considered in depression management (15). However, due to limited number of included studies, we were unable to consider these factors in our meta-analysis. Additionally, the marginal effects observed should be interpreted with caution.

In conclusion, vitamin D supplementation in older adults was not associated with an improvement in depressive symptoms. High-dose vitamin D showed a tendency of improving depressive symptoms, although the effect size and significance were limited. Further studies on the older population are necessary to evaluate the efficacy of vitamin D in a conclusive manner. Standardized methodologies should be implemented in future RCTs regarding vitamin D dosage, depression measurement scales, and baseline serum vitamin D levels to establish practical evidence for the improvement of depressive symptoms.

## Data availability statement

The original contributions presented in the study are included in the article/Supplementary material, further inquiries can be directed to the corresponding authors.

## Ethics statement

Ethical review and approval was not required for the study on human participants in accordance with the local legislation and institutional requirements. Written informed consent for participation was not required for this study in accordance with the national legislation and the institutional requirements.

## Author contributions

YP contributed to the study design, data analysis and interpretation, and manuscript writing. Y-MA and YMY contributed to the study conceptualization, interpretation of data, critical revision of the manuscript, and supervision of the study. All authors contributed to the article and approved the submitted version.

## Conflict of interest

The authors declare that the research was conducted in the absence of any commercial or financial relationships that could be construed as a potential conflict of interest.

## Publisher’s note

All claims expressed in this article are solely those of the authors and do not necessarily represent those of their affiliated organizations, or those of the publisher, the editors and the reviewers. Any product that may be evaluated in this article, or claim that may be made by its manufacturer, is not guaranteed or endorsed by the publisher.

## Abbreviations

CI: confidence interval
IU: international units
ng/mL: nanogram per milliliters
RCT: randomized controlled trial
SDs: standard deviations
SMD: standardized mean difference
VDRs: vitamin D receptors

## References

[1] World Health Organization. Ageing and health. (2022). Available at: https://www.who.int/news-room/fact-sheets/detail/ageing-and-health

[2] Zenebe Y Akele B Ws M Necho M. Prevalence and determinants of depression among old age: a systematic review and Meta-analysis. Ann General Psychiatry. (2021) 20:55. doi: 10.1186/s12991-021-00375-x, PMID: 34922595 PMC8684627

[3] World Health Organization. Mental health of older adults. (2017). Available at: https://www.who.int/news-room/fact-sheets/detail/mental-health-of-older-adults

[4] Rodda J Walker Z Carter J. Depression in older adults. BMJ. (2011) 343:d5219. doi: 10.1136/bmj.d5219 21957206

[5] Sözeri-Varma G. Depression in the elderly: clinical features and risk factors. Aging Dis. (2012) 3:465–71. 23251852 PMC3522513

[6] Birrer R Vemuri S. Depression in later life: a diagnostic and therapeutic challenge. Am Fam Physician. (2004) 69:2375–82. PMID: 15168957

[7] Cooper C Katona C Lyketsos K Blazer D Brodaty H Rabins P. A systematic review of treatments for refractory depression in older people. Am J Psychiatry. (2011) 168:681–8. doi: 10.1176/appi.ajp.2011.10081165, PMID: 21454919

[8] Eyles DW Smith S Kinobe R Hewison M McGrath JJ. Distribution of the vitamin D receptor and 1 alpha-hydroxylase in human brain. J Chem Neuroanat. (2005) 29:21–30. doi: 10.1016/j.jchemneu.2004.08.006, PMID: 15589699

[9] Bikle DD. Extraskeletal actions of vitamin D. Ann N Y Acad Sci. (2016) 1376:29–52. doi: 10.1111/nyas.13219, PMID: 27649525 PMC5031366

[10] Casseb GAS Kaster MP Rodrigues ALS. Potential role of vitamin D for the Management of Depression and Anxiety. CNS Drugs. (2019) 33:619–37. doi: 10.1007/s40263-019-00640-4, PMID: 31093951

[11] Geng C Shaikh AS Han W Chen D Guo Y Jiang P. Vitamin D and depression: mechanisms, determination and application. Asia Pac J Clin Nutr. (2019) 28:689–94. doi: 10.6133/apjcn.201912_28(4).0003, PMID: 31826364

[12] Irandoust K Taheri M. The effect of vitamin D supplement and indoor vs outdoor physical activity on depression of obese depressed women. Asian J Sports Med. (2017) 8:13311. doi: 10.5812/asjsm.13311

[13] Albuloshi T Dimala CA Kuhnle GGC Bouhaimed M Dodd GF Spencer JPE. The effectiveness of vitamin D supplementation in reducing depressive symptoms: a systematic review and Meta-analysis of randomized controlled trials (Rcts). Nutr Healthy Aging. (2022) 6:301–18. doi: 10.3233/nha-200094

[14] Sabir MS Haussler MR Mallick S Kaneko I Lucas DA Haussler CA. Optimal vitamin D spurs serotonin: 1,25-Dihydroxyvitamin D represses serotonin reuptake transport (Sert) and degradation (Mao-a) gene expression in cultured rat serotonergic neuronal cell lines. Genes Nutr. (2018) 13:19. doi: 10.1186/s12263-018-0605-7, PMID: 30008960 PMC6042449

[15] Meehan M Penckofer S. The role of vitamin D in the aging adult. J Aging Gerontol. (2014) 2:60–71. doi: 10.12974/2309-6128.2014.02.02.1, PMID: 25893188 PMC4399494

[16] Bendik I Friedel A Roos FF Weber P Eggersdorfer M. Vitamin D: a critical and essential micronutrient for human health. Front Physiol. (2014) 5:248. doi: 10.3389/fphys.2014.00248, PMID: 25071593 PMC4092358

[17] Briggs R McCarroll K O'Halloran A Healy M Kenny RA Laird E. Vitamin D deficiency is associated with an increased likelihood of incident depression in community-dwelling older adults. J Am Med Dir Assoc. (2019) 20:517–23. doi: 10.1016/j.jamda.2018.10.006, PMID: 30470577

[18] Hoogendijk W Lips P Dik M Atf B Bwjh P. Depression is associated with decreased 25-Hydroxyvitamin D and increased parathyroid hormone levels in older adults. Arch Gen Psychiatry. (2008) 65:508–12. doi: 10.1001/archpsyc.65.5.508, PMID: 18458202

[19] Li G Mbuagbaw L Samaan Z Falavigna M Zhang S Adachi JD. Efficacy of vitamin D supplementation in depression in adults: a systematic review. J Clin Endocrinol Metab. (2014) 99:757–67. doi: 10.1210/jc.2013-3450, PMID: 24423304 PMC5112012

[20] Gowda U Mutowo MP Smith BJ Wluka AE Renzaho AMN. Vitamin D supplementation to reduce depression in adults: Meta-analysis of randomized controlled trials. Nutrition. (2015) 31:421–9. doi: 10.1016/j.nut.2014.06.017, PMID: 25701329

[21] Omidian M Mahmoudi M Abshirini M Eshraghian MR Javanbakht MH Zarei M. Effects of vitamin D supplementation on depressive symptoms in type 2 diabetes mellitus patients: randomized placebo-controlled double-blind clinical trial. Diabetes Metab Syndr. (2019) 13:2375–80. doi: 10.1016/j.dsx.2019.06.011, PMID: 31405646

[22] Jorde R Sneve M Figenschau Y Svartberg J Waterloo K. Effects of vitamin D supplementation on symptoms of depression in overweight and obese subjects: randomized double blind trial. J Intern Med. (2008) 264:599–609. doi: 10.1111/j.1365-2796.2008.02008.x, PMID: 18793245

[23] Kjærgaard M Waterloo K Wang CE Almås B Figenschau Y Hutchinson MS. Effect of vitamin D supplement on depression scores in people with low levels of serum 25-Hydroxyvitamin D: nested case-control study and randomised clinical trial. Br J Psychiatry. (2012) 201:360–8. doi: 10.1192/bjp.bp.111.104349, PMID: 22790678

[24] Okereke OI Reynolds CF 3rd Mischoulon D Chang G Vyas CM Cook NR. Effect of long-term vitamin D3 supplementation vs placebo on risk of depression or clinically relevant depressive symptoms and on change in mood scores: a randomized clinical trial. JAMA. (2020) 324:471–80. doi: 10.1001/jama.2020.10224, PMID: 32749491 PMC7403921

[25] Li H Sun D Wang A Pan H Feng W Ng CH. Serum 25-Hydroxyvitamin D levels and depression in older adults: a dose-response Meta-analysis of prospective cohort studies. Am J Geriatr Psychiatry. (2019) 27:1192–202. doi: 10.1016/j.jagp.2019.05.022, PMID: 31262683

[26] Page MJ McKenzie JE Bossuyt PM Boutron I Hoffmann TC Mulrow CD. The Prisma 2020 statement: an updated guideline for reporting systematic reviews. BMJ. (2021) 372:n71. doi: 10.1136/bmj.n71, PMID: 33782057 PMC8005924

[27] National Institutes of Health. Office of Dietary Supplement - vitamin D. U.S Department of Health and Human Services: (2022) Available at: https://ods.od.nih.gov/factsheets/VitaminD-HealthProfessional/#h5.

[28] Sterne JAC Savović J Page MJ Elbers RG Blencowe NS Boutron I. Rob 2: a revised tool for assessing risk of Bias in randomised trials. BMJ. (2019) 366:l4898. doi: 10.1136/bmj.l4898 31462531

[29] Choi J No JE Lee JY Choi SA Chung WY Ah YM. Efficacy and safety of clinically driven low-dose treatment with direct Oral anticoagulants in Asians with atrial fibrillation: a systematic review and Meta-analysis. Cardiovasc Drugs Ther. (2022) 36:333–45. doi: 10.1007/s10557-021-07171-5, PMID: 33725229

[30] Borenstein M Hedges LV Higgins JPT Rothstein HR. Introdcution to Meta-analysis. New Jersey: John Wiley & Sons (2009). 21–32.

[31] Yagiz G Akaras E Kubis H-P Owen JA. The effects of resistance training on architecture and volume of the upper extremity muscles: a systematic review of randomised controlled trials and Meta-analyses. Appl Sci. (2022) 12:1593. doi: 10.3390/app12031593

[32] Higgins JPT Li T Deeks JJ. Chapter 6: Choosing effect measures and computing estimates of effect In: Higgins JPT Thomas J Chandler J Cumpston M Li T Page MJ, editors. Cochrane Handbook for Systematic Reviews of Interventions version 6.3 (updated February 2022). Cochrane, (2022). Available at: www.training.cochrane.org/handbook

[33] Hahn J Jo Y Yoo SH Shin J Yu YM Ah YM. Risk of major adverse events associated with Gabapentinoid and opioid combination therapy: a systematic review and Meta-analysis. Front Pharmacol. (2022) 13:1009950. doi: 10.3389/fphar.2022.1009950, PMID: 36304170 PMC9593000

[34] Higgins JP Thompson SG. Quantifying heterogeneity in a Meta-analysis. Stat Med. (2002) 21:1539–58. doi: 10.1002/sim.1186, PMID: 12111919

[35] Alavi NM Khademalhoseini S Vakili Z Assarian F. Effect of vitamin D supplementation on depression in elderly patients: a randomized clinical trial. Clin Nutr. (2019) 38:2065–70. doi: 10.1016/j.clnu.2018.09.011, PMID: 30316534

[36] de Koning EJ Lips P Penninx BWJH Elders PJM Heijboer AC den Heijer M. Vitamin D supplementation for the prevention of depression and poor physical function in older persons: the D-Vitaal study, a randomized clinical trial. Am J Clin Nutr. (2019) 110:1119–30. doi: 10.1093/ajcn/nqz141, PMID: 31340012 PMC6821546

[37] Dumville JC Miles JN Porthouse J Cockayne S Saxon L King C. Can vitamin D supplementation prevent winter-time blues? A randomised trial among older women. J Nutr Health Aging. (2006) 10:151–3. PMID: 16554952

[38] Sanders KM Stuart AL Williamson EJ Jacka FN Dodd S Nicholson G. Annual high-dose vitamin D₃ and mental well-being: randomised controlled trial. Br J Psychiatry. (2011) 198:357–64. doi: 10.1192/bjp.bp.110.087544 21525520

[39] Yalamanchili V Gallagher JC. Treatment with hormone therapy and calcitriol did not affect depression in older postmenopausal women: No interaction with estrogen and vitamin D receptor genotype polymorphisms. Menopause. (2012) 19:697–703. doi: 10.1097/gme.0b013e31823bcec5, PMID: 22205149 PMC3319337

[40] Zajac IT Barnes M Cavuoto P Wittert G Noakes M. The effects of vitamin D-enriched mushrooms and vitamin D3 on cognitive performance and mood in healthy elderly adults: a randomised, double-blinded, Placebo-Controlled Trial. Nutrients. (2020) 12:1–16. doi: 10.3390/nu12123847, PMID: 33339304 PMC7766163

[41] Zaromytidou E Koufakis T Dimakopoulos G Drivakou D Konstantinidou S Rakitzi P. Vitamin D alleviates anxiety and depression in elderly people with prediabetes: a randomized controlled study. Meta. (2022) 12:884. doi: 10.3390/metabo12100884, PMID: 36295786 PMC9611739

[42] Holick MF Binkley NC Bischoff-Ferrari HA Gordon CM Hanley DA Heaney RP. Evaluation, treatment, and prevention of vitamin D deficiency: an Endocrine Society clinical practice guideline. J Clin Endocrinol Metab. (2011) 96:1911–30. doi: 10.1210/jc.2011-0385, PMID: 21646368

[43] Menon V Kar SK Suthar N Nebhinani N. Vitamin D and depression: a critical appraisal of the evidence and future directions. Indian J Psychol Med. (2020) 42:11–21. doi: 10.4103/IJPSYM.IJPSYM_160_19, PMID: 31997861 PMC6970300

[44] Andrade JM Grandoff PG Schneider ST. Vitamin D intake and factors associated with self-reported vitamin D deficiency among us adults: a 2021 cross-sectional study. Front Nutr. (2022) 9:899300. doi: 10.3389/fnut.2022.899300, PMID: 35634404 PMC9131078

[45] Holick M. Vitamin D: a D-Lightful solution for health. J Investig Med. (2011) 59:872–80. doi: 10.231/JIM.0b013e318214ea2d, PMID: 21415774 PMC3738435

[46] Heaney RP. Guidelines for optimizing design and analysis of clinical studies of nutrient effects. Nutr Rev. (2014) 72:48–54. doi: 10.1111/nure.12090, PMID: 24330136

[47] Spedding S. Vitamin D and depression: a systematic review and Meta-analysis comparing studies with and without biological flaws. Nutrients. (2014) 6:1501–18. doi: 10.3390/nu6041501, PMID: 24732019 PMC4011048

