# Supplementary material for: Vitamin D supplementation for depression in older adults: a meta-analysis of randomized controlled trials
Source: Front Nutr. 2023 Jun 21;10:1169436. doi: 10.3389/fnut.2023.1169436 (PMC10320579; doi:10.3389/fnut.2023.1169436)
Supplement: Supplementary file 1 [file Data_Sheet_1.docx]

**Vitamin D Supplementation for Depression in Geriatrics: A Systematic Review and Meta-analysis**

Supplemental materials

**Supplementary Table 1.** Checklist for Preferred Reporting Items for Systematic Reviews and Meta-Analyses Flow chart of the study selection process

**Supplementary Table 2.** Search strategies

**Supplementary Table 3.** Detailed information on the risk of bias assessment according to the Risk of Bias 2 criteria.

**Supplementary Figure 1.** Quality assessment of the risk of bias according to the Risk of Bias 2 criteria.

**Supplementary Table 1.** Checklist for Preferred Reporting Items for Systematic Reviews and Meta-Analyses Flow chart of the study selection process

| **Section/topic** | **#** | **Checklist item** | **Reported on page #** |
| --- | --- | --- | --- |
| **TITLE** |  |  |  |
| Title | 1 | Identify the report as a systematic review, meta-analysis, or both. | 1 |
| **ABSTRACT** |  | |  |
| Structured summary | 2 | Provide a structured summary including, as applicable: background; objectives; data sources; study eligibility criteria, participants, and interventions; study appraisal and synthesis methods; results; limitations; conclusions and implications of key findings; systematic review registration number. | 2 |
| **INTRODUCTION** |  |  |  |
| Rationale | 3 | Describe the rationale for the review in the context of what is already known. | 3 |
| Objectives | 4 | Provide an explicit statement of questions being addressed with reference to participants, interventions, comparisons, outcomes, and study design (PICOS). | 4 |
| **METHODS** | | | |
| Protocol and registration | 5 | Indicate if a review protocol exists, if and where it can be accessed (e.g., Web address), and, if available, provide registration information including registration number. | 4 |
| Eligibility criteria | 6 | Specify study characteristics (e.g., PICOS, length of follow-up) and report characteristics (e.g., years considered, language, publication status) used as criteria for eligibility, giving rationale. | 4 |
| Information sources | 7 | Describe all information sources (e.g., databases with dates of coverage, contact with study authors to identify additional studies) in the search and date last searched. | 4 |
| Search | 8 | Present full electronic search strategy for at least one database, including any limits used, such that it could be repeated. | Supplementary Table 2 |
| Study selection | 9 | State the process for selecting studies (i.e., screening, eligibility, included in systematic review, and, if applicable, included in the meta-analysis). | 4 |
| Data collection process | 10 | Describe method of data extraction from reports (e.g., piloted forms, independently, in duplicate) and any processes for obtaining and confirming data from investigators. | 5 |
| Data items | 11 | List and define all variables for which data were sought (e.g., PICOS, funding sources) and any assumptions and simplifications made. | 4 |
| Risk of bias in individual studies | 12 | Describe methods used for assessing risk of bias of individual studies (including specification of whether this was done at the study or outcome level), and how this information is to be used in any data synthesis. | Supplementary Table 3 |
| Summary measures | 13 | State the principal summary measures (e.g., risk ratio, difference in means). | 5 – 6 |
| Synthesis of results | 14 | Describe the methods of handling data and combining results of studies, if done, including measures of consistency (e.g., I^2^) for each meta-analysis. | 5 – 6 |
| Risk of bias across studies | 15 | Specify any assessment of risk of bias that may affect the cumulative evidence (e.g., publication bias, selective reporting within studies). | 5 – 6 |
| Additional analyses | 16 | Describe methods of additional analyses (e.g., sensitivity or subgroup analyses, meta-regression), if done, indicating which were pre-specified. | 6 |
| **RESULTS** | | | |
| Study selection | 17 | Give numbers of studies screened, assessed for eligibility, and included in the review, with reasons for exclusions at each stage, ideally with a flow diagram. | 6 |
| Study characteristics | 18 | For each study, present characteristics for which data were extracted (e.g., study size, PICOS, follow-up period) and provide the citations. | 6 |
| Risk of bias within studies | 19 | Present data on risk of bias of each study and, if available, any outcome level assessment (see item 12). | 7 |
| Results of individual studies | 20 | For all outcomes considered (benefits or harms), present, for each study: (a) simple summary data for each intervention group (b) effect estimates and confidence intervals, ideally with a forest plot. | 6 – 7 |
| Synthesis of results | 21 | Present results of each meta-analysis done, including confidence intervals and measures of consistency. | 6 – 7 |
| Risk of bias across studies | 22 | Present results of any assessment of risk of bias across studies (see Item 15). | Supplementary Figure 1 |
| Additional analysis | 23 | Give results of additional analyses, if done (e.g., sensitivity or subgroup analyses, meta-regression [see Item 16]). | 7 |
| **DISCUSSION** |  |  |  |
| Summary of evidence | 24 | Summarize the main findings including the strength of evidence for each main outcome; consider their relevance to key groups (e.g., healthcare providers, users, and policy makers). | 7 – 9 |
| Limitations | 25 | Discuss limitations at study and outcome level (e.g., risk of bias), and at review-level (e.g., incomplete retrieval of identified research, reporting bias). | 8 |
| Conclusions | 26 | Provide a general interpretation of the results in the context of other evidence, and implications for future research. | 8 – 9 |
| **FUNDING** |  |  |  |
| Funding | 27 | Describe sources of funding for the systematic review and other support (e.g., supply of data); role of funders for the systematic review. | 9 |

**Supplementary Table 2.** Search strategy

| **PubMed** | |
| --- | --- |
| **No** | **Query** |
| #1 | "aged"[MeSH Terms] OR "elder*"[Text Word] OR "oldest old"[Text Word] OR "nonagenarian*"[Text Word] OR "octogenarian*"[Text Word] OR "centenarian*"[Text Word] OR "septuagenarian*"[Text Word] OR "geriatric*"[Text Word] OR "geriatrician*"[Text Word] OR "Seniors"[Text Word] OR "Senior"[Text Word] OR "older people"[Text Word] OR "older patient*"[Text Word] OR "older person*"[Text Word] OR "older women"[Text Word] OR "older men"[Text Word] OR "older individual*"[Text Word] OR "older adult*"[Text Word] OR "older adulthood"[Text Word] OR "Aging"[Text Word] OR "later life"[Text Word] |
| #2 | "vitamin d"[MeSH Terms] OR "ergocalciferols"[MeSH Terms] OR "vitamin d*"[Text Word] OR "cholecalciferol*"[Text Word] OR "ergocalciferol*"[Text Word] OR "Calciol"[Text Word] OR "calciferol*"[Text Word] OR "d2 vitamin"[Text Word] |
| #3 | "depressive disorder"[MeSH Terms] OR "depression"[MeSH Terms] OR "depressive disorder, major"[MeSH Terms] OR "involutional psychoses"[Text Word] OR "involutional psychosis"[Text Word] OR "involutional melancholia"[Text Word] OR "depressi*"[Text Word] |
| #4 | ("randomized controlled trial"[Publication Type] OR "controlled clinical trial"[Publication Type] OR "randomized"[Title/Abstract] OR "placebo"[Title/Abstract] OR "drug therapy"[MeSH Subheading] OR "randomly"[Title/Abstract] OR "trial"[Title/Abstract] OR "groups"[Title/Abstract]) NOT ("animals"[MeSH Terms] NOT "humans"[MeSH Terms]) |
| #5 | #1 AND #2 AND #3 AND #4 |
| **EMBASE** | |
| #1 | ‘Aged’/exp OR aged OR ‘Geriatric’/exp OR ‘Geriatrics’/exp OR ‘Elder*’ OR ‘Oldest Old’ OR ‘Nonagenarian*’ OR ‘Octogenarian*’ OR ‘Centenarian*’ OR ‘Septuagenarian*’ OR ‘Geriatric*’ OR ‘Seniors’ OR ‘Senior’ OR ‘Older people’ OR ‘Older patient*’ OR ‘Older person*’ OR ‘Older women’ OR ‘Older men’ OR ‘Older individual*’ OR ‘Older adult*’ OR ‘Aging’ OR ‘later life’ |
| #2 | ‘VitaminD’/exp OR ‘vitamin D’ OR 'vitamin d2' OR 'vitamin d3' OR 'al*acalcidol' OR 'c?olecalciferol' OR 'calcitriol' OR 'calcidiol' OR 'calcifediol' OR 'calciferol' OR 'calciol' OR 'calderol' OR 'dedrogyl' OR 'dihydrotachysterol' OR 'dihydroxycolecalciferol' OR 'dihydroxycholecalciferol' OR 'dihydroxyvitamin d' OR 'dihydroxyvitamin d2' OR 'dihydroxyvitamin d3' OR 'doxercalciferol' OR 'eldecalcitol' OR 'ercalcidiol' OR 'ergocalciferol*' OR 'hidroferol' OR 'hydroxycalciferol' OR 'hydroxylcalciferol' OR 'hydroxycolecalciferol' OR 'hydroxycholecalciferol' OR 'hydroxyergocalciferol*' OR 'hydroxyvitamin d' OR 'hydroxyvitamin d2' OR 'hydroxyvitamin d3' OR 'paricalcitol' |
| #3 | ‘depression’/exp OR ‘involutional psychosis’/exp OR ‘sadness’/exp OR Depressi* OR dysphoria OR dysthymia OR melancho* OR mourning OR sadness |
| #4 | ‘randomzied controlled trial’/exp OR ‘randomized controlled trial’ OR 'crossover procedure':de OR 'double-blind procedure':de OR 'randomized controlled trial':de OR 'single-blind procedure':de OR (random* OR factorial* OR crossover* OR cross NEXT/1 over* OR placebo* OR doubl* NEAR/1 blind* OR singl* NEAR/1 blind* OR assign* OR allocat* OR volunteer*):de,ab,ti |
| #5 | ('animal':de OR 'animal experiment':de OR 'nonhuman':de) NOT ('human':de OR 'human experiment':de) |
| #6 | #4 NOT #5 |
| #7 | #1 AND #2 AND #3 AND #6 |
| **CENTRAL** | |
| #1 | MeSH descriptor: [Aged] explode all trees |
| #2 | Elder* OR Oldest old OR Nonagenarian* OR Octogenarian* OR Centenarian* OR Septuagenarian* OR Geriatric* OR Geriatrician* OR Seniors OR Senior OR Older people OR Older patient* OR Older person* OR Older women OR Older men OR Older individual* OR Older adult* OR Older adulthood OR Aging OR 'later life' |
| #3 | #1 OR #2 |
| #4 | MeSH descriptor: [Vitamin D] explode all trees |
| #5 | “Vitamin D*” OR Cholecalciferol* OR Ergocalciferol* OR Calciol OR Calciferol* OR “D2, vitamin” |
| #6 | #4 OR #5 |
| #7 | MeSH descriptor: [Depression] explode all trees |
| #8 | MeSH descriptor: [Depressive Disorder, Major] explode all trees |
| #9 | Depressi* OR Involutional Psychoses OR Involutional Psychosis OR Involutional Melancholia |
| #10 | #7 OR #8 OR #9 |
| #11 | #3 AND #6 AND #10 |
| **PsychInfo** | |
| #1 | MAINSUBJECT.EXACT.EXPLODE("Older Adulthood") OR MAINSUBJECT.EXACT.EXPLODE("Geriatric Patients") OR MAINSUBJECT.EXACT.EXPLODE("Geriatrics") OR MAINSUBJECT.EXACT.EXPLODE("Aged (Attitudes Toward)") OR Elder* OR Oldest Old OR Nonagenarian* OR Octogenarian* OR Centenarian* OR Septuagenarian* Seniors OR Senior OR Older people OR Older patient* OR Older person* OR Older women OR Older men OR Older individual* OR Older adult* OR Aging OR 'later life' |
| #2 | 'vitamin d2' OR 'vitamin d3' OR 'al*acalcidol' OR 'c?olecalciferol' OR 'calcitriol' OR 'calcidiol' OR 'calcifediol' OR 'calciferol' OR 'calciol' OR 'calderol' OR 'dedrogyl' OR 'dihydrotachysterol' OR 'dihydroxycolecalciferol' OR 'dihydroxycholecalciferol' OR 'dihydroxyvitamin d' OR 'dihydroxyvitamin d2' OR 'dihydroxyvitamin d3' OR 'doxercalciferol' OR 'eldecalcitol' OR 'ercalcidiol' OR 'ergocalciferol*' OR 'hidroferol' OR 'hydroxycalciferol' OR 'hydroxylcalciferol' OR 'hydroxycolecalciferol' OR 'hydroxycholecalciferol' OR 'hydroxyergocalciferol*' OR 'hydroxyvitamin d' OR 'hydroxyvitamin d2' OR 'hydroxyvitamin d3' OR 'paricalcitol' |
| #3 | MAINSUBJECT.EXACT.EXPLODE("Major Depression") OR MAINSUBJECT.EXACT.EXPLODE("Late Life Depression") OR MAINSUBJECT.EXACT.EXPLODE("Depression (Emotion)") OR depressive Or dysphoria OR dysthymia OR melancho* OR mourning |
| #4 | SU.EXACT("Treatment Effectiveness Evaluation") OR SU.EXACT.EXPLODE("Treatment Outcomes") OR SU.EXACT("Placebo") OR SU.EXACT("Followup Studies") OR placebo* OR random* OR "comparative stud*" OR  clinical NEAR/3 trial* OR research NEAR/3 design OR evaluat* NEAR/3 stud* OR prospectiv* NEAR/3 stud* OR (singl* OR doubl* OR trebl* OR tripl*) NEAR/3 (blind* OR mask*) |
| #5 | #1 AND #2 AND #3 AND #4 |

**Supplementary Table 3.** Detailed information on the risk of bias assessment according to the Risk of Bias 2 criteria.

| **Study**  **(author, year)** | **The risk of bias domain** | **Assessment of risk of bias** | **The reason for judgement** |
| --- | --- | --- | --- |
| Alavi, 2019 | Randomization process | Low risk | Computer-generated random assignment and allocation were concealed from the researcher and participants. |
|  | Deviations from intended intervention | Low risk | Allocation was concealed from the researcher and participants until the analyses were completed. No probable deviations from intended intervention were found due to the trial context. |
|  | Missing outcome data | Low risk | Outcome data were available for all participants except for one participant in each group. |
|  | Measurement of the outcome | Low risk | The measurement of the outcome did not differ between intervention groups. |
|  | Selection of the reported result | Low risk | All results were reported in pre-specified manners. |
|  | Overall | Low risk |  |
| De Koning 2019 | Randomization process | Low risk | Randomization is processed by an independent pharmacist in 1:1 ratio in blocks of 4. No notable baseline imbalance between the two groups. |
|  | Deviations from intended intervention | Low risk | Allocation was concealed from the participants, researchers, and research nurses. Therefore, no probable deviations from intended intervention were found. |
|  | Missing outcome data | Low risk | Nearly all outcomes were available except for 4 participants who had to be excluded. |
|  | Measurement of the outcome | Low risk | Appropriate outcome measurement without differences in between the intervention groups. |
|  | Selection of the reported result | Low risk | All pre-specified outcome measurements were reported in a pre-specified manner. |
|  | Overall | Low risk |  |
| Dumville 2006 | Randomization process | Low risk | Independent researchers not involved in the study conducted blinded randomization of eligible participants. Multiple block sizes were used for randomization. No notable baseline imbalance between the two groups. |
|  | Deviations from intended intervention | Low risk | Operating physicians and nurses were aware of the intervention group because the participants had to visit the nurses to get 6 months of supply. No probable deviations from intended intervention were found. |
|  | Missing outcome data | High risk | Only 77% of participants had a valid SF-12 score at both baseline and after 6 months. The missing data is large and the outcome may depend on its true value. |
|  | Measurement of the outcome | Low risk | Appropriate outcome measurement without differences in between the intervention groups. |
|  | Selection of the reported result | Low risk | All pre-specified outcome measurements were reported in a pre-specified manner. |
|  | Overall | High risk |  |
| Sanders 2011 | Randomization process | Low risk | Double-blinded, computer randomization of participants’ study ID number in which the participants and study staff were masked to the treatment allocation. No notable differences were found between intervention groups. |
|  | Deviations from intended intervention | Low risk | Allocation to the treatment was masked in both to participants and study staff until the completion of the study. |
|  | Missing outcome data | Low risk | Nearly all participants (99%) completed the SF-12. |
|  | Measurement of the outcome | Low risk | Appropriate outcome measurement without differences in between the intervention groups. |
|  | Selection of the reported result | Low risk | All pre-specified outcome measurements were reported in a pre-specified manner. |
|  | Overall | Low risk |  |
| Yalamanchili 2012 | Randomization process | Low risk | Double-blind, placebo-controlled intervention study was conducted without notable differences between the two groups. Computer-generated randomization into four groups. |
|  | Deviations from intended intervention | Low risk | No probable deviation from the intended intervention may exist due to the context of the trial. |
|  | Missing outcome data | Low risk | Only 85% of participants completed the GDS questionnaire therefore the author conducted modified intent-to-treat analysis. |
|  | Measurement of the outcome | Low risk | Recall bias may be presented because depression was self-reported instead of clinically diagnosed. No difference in outcome measurement in two groups. |
|  | Selection of the reported result | Low risk | All pre-specified outcome measurements were reported in a pre-specified manner. |
|  | Overall | Low risk |  |
| Zajac 2020 | Randomization process | Low risk | Double-blinded, placebo-controlled, randomized through computer-based minimization process with some differences in between the group but not significant. |
|  | Deviations from intended intervention | Low risk | Opaque containers were used to mask allocation to ensure blinding of researchers and participants. No probable deviations from intended intervention may exist due to the context of the trial. |
|  | Missing outcome data | Low risk | The outcome of 2015 cohort were available for analysis; however, 2014 cohort had to be excluded due to underdose of the treatment group. |
|  | Measurement of the outcome | Low risk | Appropriate outcome measurement was used to analyze the data without the difference in between the intervention groups. |
|  | Selection of the reported result | Low risk | All pre-specified outcome measurements were reported in a pre-specified manner. |
|  | Overall | Low risk |  |
| Zaromytidou 2022 | Randomization process | High risk | Open-label design with randomly assigned by computer code. No significant difference in between the two groups were detected. |
|  | Deviations from intended intervention | Low risk | Higher dropout rate in placebo compared to vitamin D group. No evidence of possible deviation from intended intervention. |
|  | Missing outcome data | Low risk | Only 85.6% completed the trial but no evidence was available to detect bias due to missing data. |
|  | Measurement of the outcome | Low risk | Appropriate outcome measurement without differences in between the intervention groups. |
|  | Selection of the reported result | Low risk | All pre-specified outcome measurements were reported in a pre-specified manner. |
|  | Overall | High risk |  |

**Supplementary Figure 1.** Quality assessment of the risk of bias according to the Risk of Bias 2 criteria.
